# How will the main risk factors contribute to the burden of non-communicable diseases under different scenarios by 2050? A modelling study

**DOI:** 10.1371/journal.pone.0231725

**Published:** 2020-04-29

**Authors:** Marion Devaux, Aliénor Lerouge, Giovanna Giuffre, Susanne Giesecke, Sara Baiocco, Andrea Ricci, Francisco Reyes, David Cantarero, Bruno Ventelou, Michele Cecchini

**Affiliations:** 1 Organisation for Economic Cooperation and Development, Paris, France; 2 ISINNOVA, Institute of Studies for the Integration of Systems, Rome, Italy; 3 Austrian Institute of Technology, Seibersdorf, Austria; 4 Centre for European Policy Studies, Brussels, Belgium; 5 Universidad de Vigo, Vigo, Spain; 6 Universidad de Cantabria, Cantabria, Spain; 7 Aix-Marseille Université, CNRS, EHESS, Centrale Marseille, IRD, AMSE, Marseille, France; British Columbia Centre for Excellence in HIV/AIDS, CANADA

## Abstract

**Background:**

The future burden of non-communicable diseases (NCDs) depends on numerous factors such as population ageing, evolution of societal trends, behavioural and physiological risk factors of individuals (e.g. smoking, alcohol use, obesity, physical inactivity, and hypertension). This study aims to assess the burden of NCDs in Europe by 2050 under alternative scenarios.

**Methods:**

This study combines qualitative and quantitative forecasting techniques to examine how population health in Europe may evolve from 2015 to 2050, taking into account future societal trends. Four scenarios were developed (one business-as-usual scenario, two response scenarios and one pessimistic scenario) and assessed against ‘best’ and ‘worst’-case scenarios. This study provides quantitative estimates of both diseases and mortality outcomes, using a microsimulation model incorporating international survey data.

**Findings:**

Each scenario is associated with a different risk factor prevalence rate across Europe during the period 2015–2050. The prevalence and incidence of NCDs consistently increase during the analysed time period, mainly driven by population ageing. In more optimistic scenarios, diseases will appear in later ages, while in the pessimistic scenarios, NCDs will impair working-age people. Life expectancy is expected to grow in all scenarios, but with differences by up to 4 years across scenarios and population groups. Premature mortality from NCDs will be reduced in more optimistic scenarios but stagnate in the worst-case scenario.

**Interpretation:**

Population ageing will have a greater impact on the spread of NCDs by 2050 compared to risk factors. Nevertheless, risk factors, which are influenced by living environments, are an important factor for determining future life expectancy in Europe.

## Introduction

In the coming decades, countries will continue to experience an increase in the burden of non-communicable diseases (NCDs), which is associated with considerable medical and non-medical costs. The cost of NCDs to healthcare systems currently accounts for almost half of the general hospital expenditures in most developed countries [1,2]. Moreover, increasing NCDs add significant cost to individuals, families, businesses, and governments–e.g. unemployment and work absence, which could potentially harm the whole economy.

A large share of NCDs are preventable through the reduction of four behavioural risk factors (RFs): tobacco use, physical inactivity, harmful use of alcohol, and unhealthy diet [3]. However, NCDs have a multifactorial etiology and result from complex interactions between individuals and the environment, including their opportunities for better health and countering vulnerability to risks. Individual characteristics and health protective factors (such as emotional resilience), together with social, economic and environmental factors determine differences in exposure and vulnerability of individuals to health-compromising conditions [4]. Equity is also important since vulnerable and socially disadvantaged people tend to lead less-healthy lifestyles, have worse health and die earlier due to NCDs compared to people with a higher socioeconomic status [5]. Hence, the future health outlook will depend heavily on how major societal trends–e.g. migration, technology, economy, urbanisation, climate change, agriculture, citizen empowerment, and equity–improve or deteriorate peoples’ living environment, and how this may increase, or mitigate, the growth of NCDs.

There exists a variety of techniques, both qualitative and quantitative, to estimate future population health outcomes. Modelling studies are among the most sophisticated and are prominent in the literature. Modelling studies offer the capacity to project future outcomes based on historical trends or hypothetical scenarios. The literature includes recent modelling studies, which, in general, aim to forecast the impact of reducing key RFs on mortality to meet WHO NCD targets [6] and the Sustainable Development Goals (SDGs) [7]. These studies look at a variety of outcomes categorised into two broad groups: either predicted premature mortality [8,9,10] or the burden of diseases [11,12,13]. Three studies report on health projections based on alternative scenarios [9,10,13], while one study displays results of various policy interventions [12]. The influence of broader societal trends is included in one study [13].

This study adds to the current literature by using qualitative and quantitative forecasting techniques to estimate future population health in Europe by 2050, which takes into account major societal trends (i.e. migration, technology, economy, urbanisation, climate change, agriculture, citizen empowerment, and equity). Furthermore, it provides quantitative estimates of future health outcomes including disabilities and healthy life years.

The main objective of this study is to assess the burden of NCDs in European countries under alternative scenarios reflecting major societal trends between 2015 and 2050. To achieve this, the study combines, for the first time to our knowledge, qualitative scenario building techniques with quantitative microsimulation modelling. It also quantifies the contribution of the determinants of NCDs to inform the policy-making debate regarding suitable actions to contain the future burden of NCDs.

## Methods

### Study setting

This study focusses on European countries categorised in three regions (Central-Eastern, Northern and Southern Europe) (country grouping in S2 Appendix). The base year is 2015, while the projection period runs from 2015 to 2050.

### Study data

Qualitative data on emerging societal trends aimed at building scenarios were collected from horizon scanning and expert feedback (S1 Appendix). Quantitative data used in the microsimulation model to project the burden of NCDs included international survey data on demography, epidemiology and risk factors, as well as data on the future level of RFs collected from expert opinion. Demographic data was sourced from the United Nations population projections [14] and the Human Mortality database [15]. Epidemiological data was sourced from the Institute of Health Metrics and Evaluation Global Burden of Diseases (IHME GBD) 2016 [16]. Finally, data for population exposure to RFs was taken from IHME GBD 2016 (for smoking and physical inactivity), IHME GBD 2015 and WHO Global Health Observatory [17] (alcohol consumption), and NCD-RisC data [18] (for obesity and blood pressure). These data sources are listed in S2 Appendix.

### FRESHER scenarios: Combining societal trends, imagining health futures

FRESHER (“FoResight and Modelling for European Health policy and Regulation”) is an interdisciplinary research project funded by the European Union’s Horizon 2020 research and innovation programme, which led to four scenarios, written in the form of storytelling narratives, describing possible impacts of the combination of identified trends on the future of health and NCDs (S1 Appendix).

The scenarios represent a range of alternative pathways for population health in Europe. Different medium- and long-term visions can therefore be used to plan policies which best address challenges associated with rising rates of NCDs. The scenarios in this study focus on the broader social determinants of health, exploring how structural changes in government policy, the economy, environment and society influence citizens’ behaviour, and consequently their health status.

A standard and formal integrated process was used to build four scenarios [19,20,21]. This process involved a horizon scanning phase and the selection of eight relevant societal trends identified as follows: equity, economic pattern and technological change, innovation in medicine, citizen empowerment, climate change and low-carbon economy, demographic change, urbanisation, agriculture and global food chains. Depending on the directions of these trends in future years, NCDs will be impacted in different ways and to different degrees.

**Fig 1:**
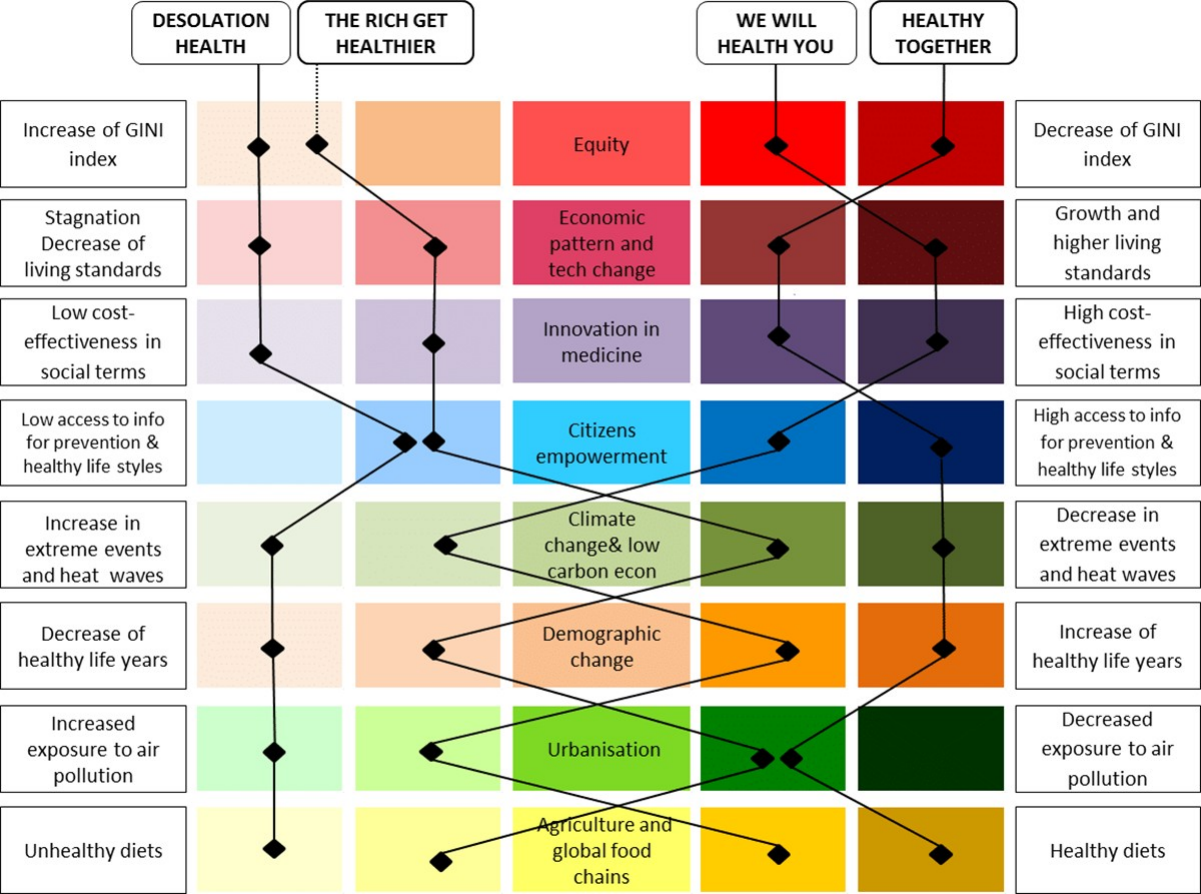
Major trends embedded in the four FRESHER scenarios. Reading note: The ‘Healthy Together’ scenario assumes that the priority is to promote health and well-being for all. In this scenario, the society is characterised by: low level of inequality (GINI index), growth and high-living standards, high cost-effectiveness in social terms, high access to information for prevention and healthy lifestyles, decrease in extreme events and heat waves, increase in healthy life years, decrease in exposure to air pollution, and healthy diets. The full narrative description of the FRESHER scenarios is available at: https://www.foresight-fresher.eu/fresher-project-results/four-fresher-scenarios/.

The process led to the development of four scenarios, representing markedly different visions of how the eight trends could evolve and influence health by 2050, as shown in Fig 1. Table 1 briefly describes the four FRESHER scenarios, including one business-as-usual scenario, two response scenarios built as results of anticipated policy responses, and one pessimistic scenario depicting how the situation could worsen. S1 Appendix provides step-wise details for creating each scenario as well as a description of the FRESHER scenarios.

**Table 1:** Description of the four FRESHER scenarios.

Scenario name	Global trend	Brief description
“The rich get healthier”	Business-as-usual scenario	Assumes a deterioration of the equity trend and the implementation of reactive climate change policies that focus on technology solutions
“We will Health you”	Optimistic future-looking response scenario	Assumes that governments and the private sector collaborate to maintain a healthy workforce and to ensure economic growth. Environmental sustainability remains a low priority as efforts are focused on producing and delivering more to everyone.
“Healthy together”	Optimistic future-looking response scenario	Assumes that governments, private sector and citizens’ networks work closely together to develop and experiment with solutions that promote quality of life, healthy opportunities and environmental sustainability
“Desolation Health”	Pessimistic future-looking scenario	Depicts how the situation could worsen with economic recession, rising inequalities and deterioration of living standards, among others

### The microsimulation model

A microsimulation model was used to predict population health outcomes by 2050 in three European macro-regions (Central-Eastern, Northern and Southern) under each scenario. This modelling platform, initially based on previous work by the Organisation for Economic Cooperation and Development (OECD) [22, 23], was adapted as part of the FRESHER project [24].

The link between the four scenarios and the model relied on five RFs (smoking, alcohol consumption, obesity, physical inactivity and high blood pressure). To forecast the burden of NCDs, a predicted evolution of RFs, as reported through a consultation among 90 health experts (S2 Appendix), was assigned to each scenario. For validation purposes, a complementary set of objective values predicting RFs was used as a comparison with expert-based values. The objective measures represented the best-case and worst-case scenarios that assumed all countries would converge to minimum (best) and maximum (worst) values of RFs as observed in 2015 in Europe.

The model was comprised of three major modules which reproduced the demography, epidemiology and RFs characteristics by age and gender-specific population groups of a given country at different points in time. The demography module simulated births, deaths, and inward/outward migration. Epidemiological characteristics of the model included disease incidence, prevalence, remission and fatality for the following groups of NCDs: diabetes, cardiovascular diseases (CVDs) (ischemic heart disease, stroke and atrial fibrillation), respiratory diseases (chronic obstructive pulmonary diseases (COPD)), neoplasms (lung, colorectal, breast and oesophageal), depression, dementia, musculoskeletal diseases (back pain, gout and rheumatoid arthritis), cirrhosis and lower respiratory infections. In addition, the model accounted for all the other causes of deaths through a residual mortality rate. RFs are associated with relative risks of developing NCDs. Details on modelling assumptions are available in S2 Appendix and in a related technical document [24].

The baseline projection of the model explores the impact of current demographic projections on the spread of NCDs by 2050, assuming no change in RFs or the probability of developing NCDs for women and men of all age groups as of 2015 in Europe. Model projections of the four alternative scenarios are compared to the baseline projection in order to estimate the expected effects on health outcomes by 2050. The health outcomes include: disease incidence, disease incidence in the working-age population, disease prevalence, comorbidity, life expectancy, and premature mortality (defined as the probability of dying between age 30 and 70 from NCDs).

## Results

### Evolution of risk factors

**Fig 2:**
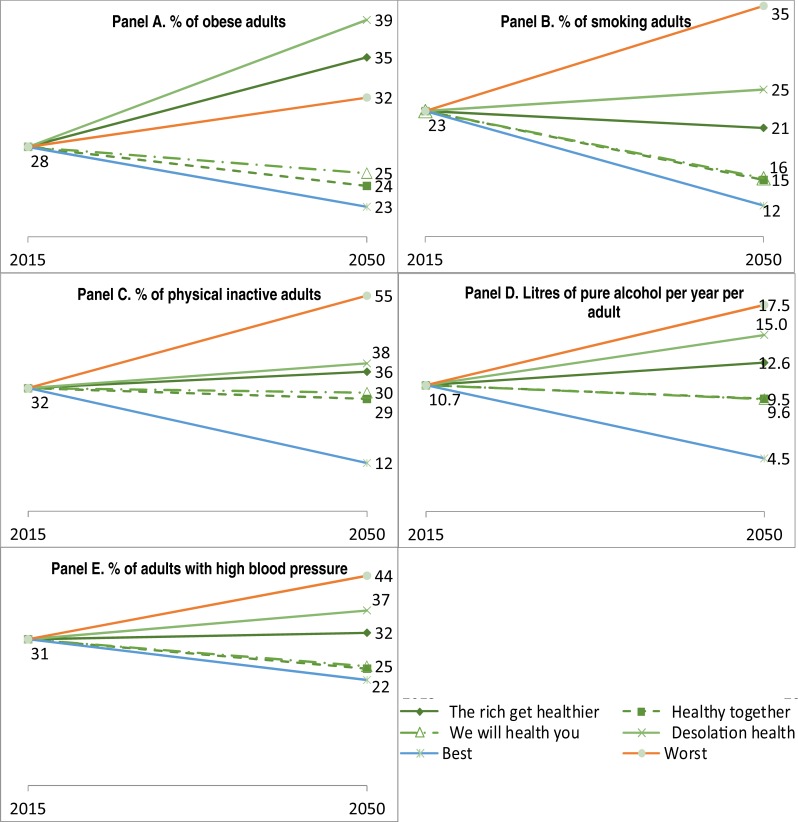
Predicted evolutions of risk factors by 2050, European countries. Source: Data source for 2015 reported in S2 Appendix, and expert’s consultation for 2050.

Levels of RFs decreased by similar amounts in two response scenarios (“Healthy Together” and “We Will Health You”) and increased in two others (“Desolation Health” and “The Rich Get Healthier”, except for smoking rates which declined marginally in the latter scenario), according to the expert opinion (Fig 2). Interestingly, all the RFs in the four scenarios based on expert predictions are between the values of the worst and best-case scenario, with the exception of obesity. Specifically, obesity rates would significantly increase to a higher level than what is observed in 2015 in Europe in two scenarios (“Desolation Health” and “The Rich Get Healthier”) (Fig 2).

### Evolution of diseases

**Fig 3:**
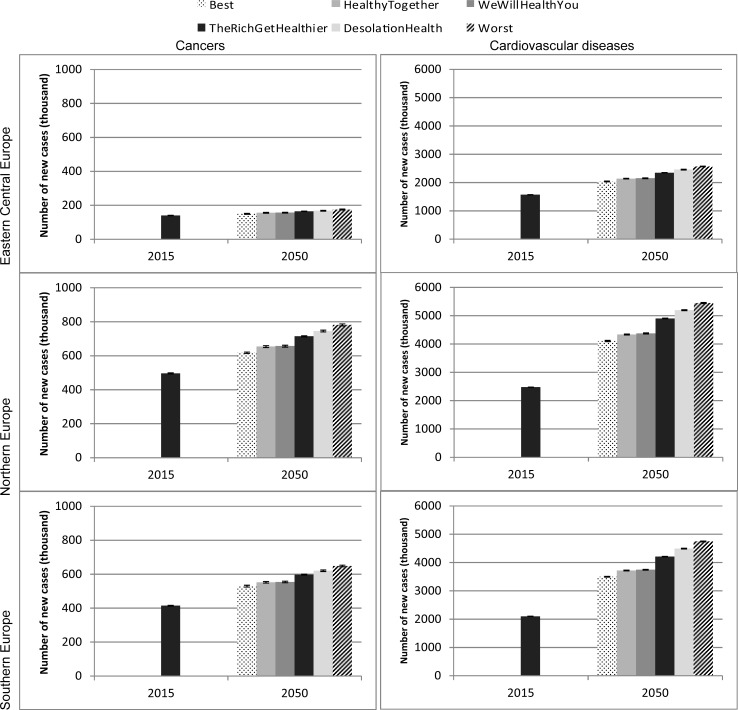
Incidence of diseases under different scenarios, 2015 and 2050, by region. Source: Authors’ estimates based on microsimulation model, April 2018. And 95% confidence intervals.

Fig 3 shows the number of new cases of cancers and CVDs in the three European regions by 2050. In ‘The Rich Get Healthier’ scenario, the number of people with cancer in Central-Eastern Europe will increase by 17% between 2015 and 2050 and by 44% in Northern and Southern Europe. The variation across scenarios is important: the incidence of cancers is 17% higher in the worst-case compared to the best-case scenario in Central-Eastern Europe (with this figure increasing to 26% and 22% in Northern Europe and Southern Europe, respectively). Similarly for CVDs, the incidence of disease will increase in all scenarios; the number of CVDs will increase by 50% in Central-Eastern Europe, by 98% in Northern Europe and by 100% in Southern Europe, with the variation across scenarios ranging from 26% to 36% depending on the region. While the results presented here focus on cancers and CVDs for clarity purposes, results for COPD and dementia show similar pattern (S1 Fig).

**Fig 4:**
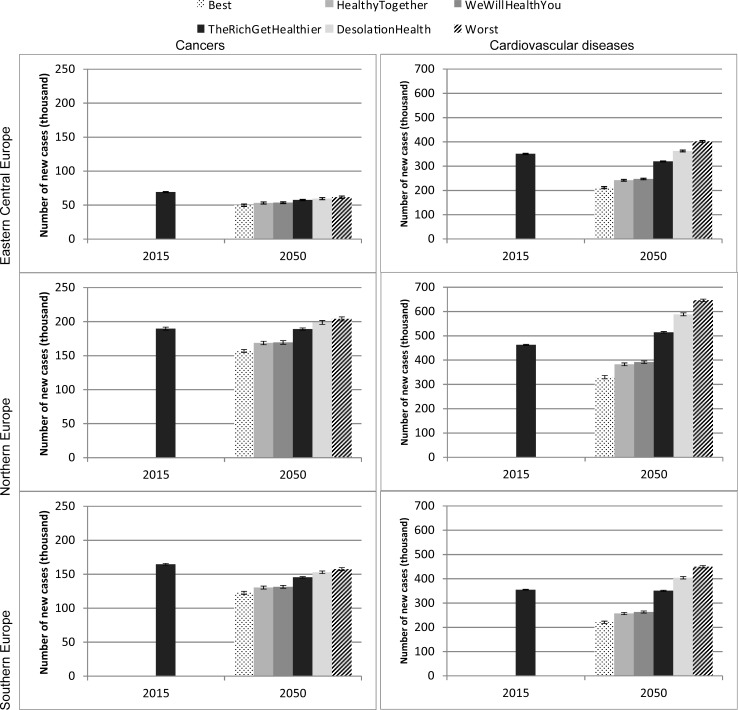
Incidence of diseases in population aged 25–65, 2015 and 2050, by region. Source: Authors’ estimates based on microsimulation model, April 2018. And 95% confidence intervals.

Looking at the working-age population aged 25–65, the incidence of diseases will decrease in optimistic scenarios (“We will Health you” and “Healthy Together”), but with differences across scenarios and regions (Fig 4). In Central-Eastern and Southern Europe, the proportion of the population aged 25–65 with cancer will decrease by 2050 in all scenarios, while the incidence of CVDs will decrease in most scenarios, with the exception of “Desolation Health” and the worst-case scenario. In Northern Europe, the incidence of cancers and CVDs in the age group 25–65 will decrease by 2050 in the two response scenarios and the best-case scenario, but will increase in “Desolation Health” and the worst-case scenario (and in “The Rich Get Healthier” for CVDs only). The size of the population aged 25–65 remains virtually unchanged and does not have an effect on variations in the disease incidence. Overall, these findings suggest that in more optimistic scenarios (two response scenarios and best-case scenario), diseases will occur later in life, whereas in more pessimistic scenarios (“Desolation Health” and worst-case scenario), the disease burden on working-age individuals will increase.

**Fig 5:**
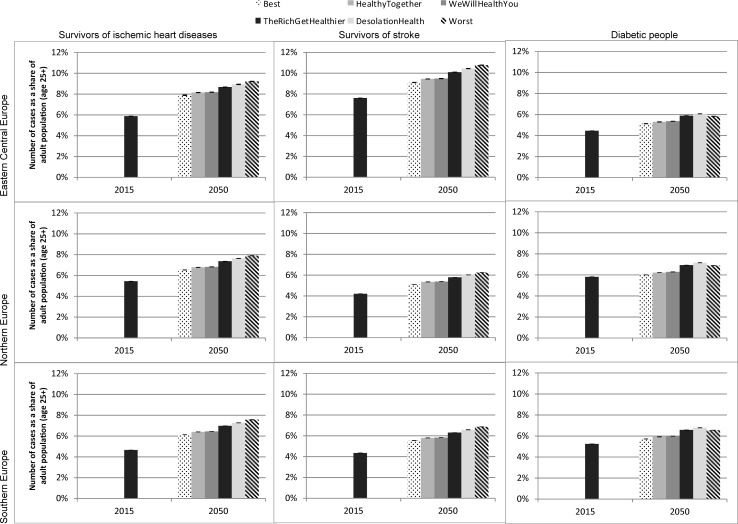
Prevalence of diseases in adult population aged 25+ under different scenarios, 2015 and 2050, by region. Source: Authors’ estimates based on microsimulation model, April 2018. And 95% confidence intervals.

The prevalence of major NCDs amongst adults will grow by 2050 as people live longer and as the proportion of elderly people in the population increases (Fig 5). In the business-as-usual scenario (“The Rich Get Healthier”), the number of people with diabetes and ischemic heart disease (IHD), as well as stroke survivors will increase in all three regions at different rates. For diabetes, the percentage growth ranges from 19% to 32%, for IHD, 35% to 50%, and for stroke survivors, 33% to 45%.

The prevalence of disease will increase, at different rates, in all scenarios. Compared to business-as-usual, the number of IHD and stroke survivors in 2050 is lower in the two response scenarios (“We will Health you” and “Healthy Together”) and the best-case scenario, while it is higher in “Desolation Health” and the worst-case scenario. For diabetes, the “The Rich Get Healthier” and “Desolation Health” scenarios exceed the worst-case scenario, in line with expert predictions on the rate of obesity.

Comorbidity is a key challenge for future elderly people. Not only does the risk of having a NCD increase with age, but so too does the number of comorbidities (S2 Fig). Results show that in Central-Eastern Europe under the business-as-usual scenario (“The Rich Get Healthier”), 19.5% of those aged 45–64 years will have one NCD by 2050. This figure increases to 35% for 65–74 year olds and 60% for those aged 75 years or over. Under the same scenario and region, the proportion of people with two or more NCDs will reach 3% for the 45–64 year age group and 25% for the oldest age group. Similar results are found for Southern and Northern Europe, with marginally lower proportions for multi-morbidities.

### Life expectancy and premature mortality

**Fig 6:**
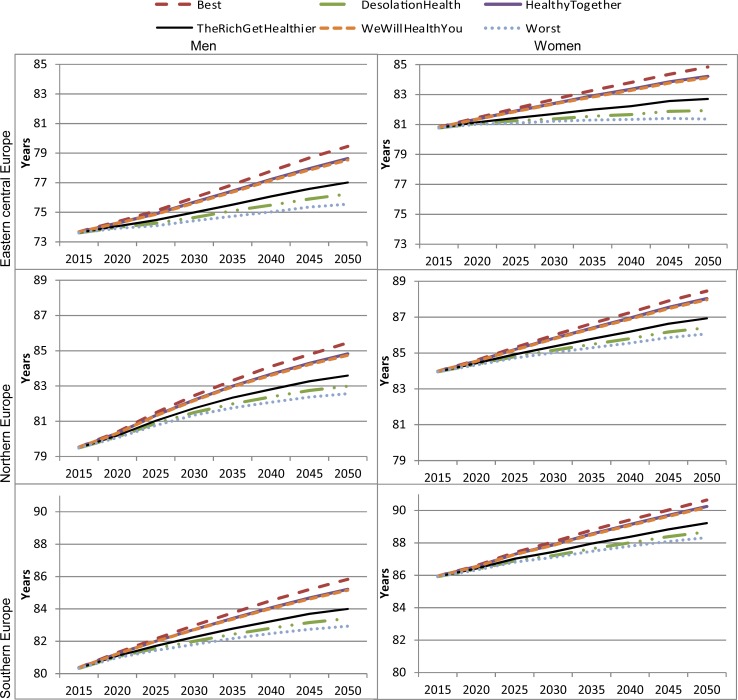
Life expectancy by 2050 under different scenarios, by gender and region. Source: Authors’ estimates based on microsimulation model, April 2018.

All projections show gains in life expectancy between 2015 and 2050, with differences across regions and scenarios by up to 4 years (Fig 6). In the business-as-usual scenario (‘The Rich Get Healthier’), male (female) life expectancy is predicted to grow to 77.0 (82.7) years in Central-Eastern Europe, to 83.6 (86.9) years in Northern Europe and 84.0 (89.2) years in Southern Europe. Life expectancy gains vary significantly across scenarios. In Central-Eastern Europe, gains in male life expectancy vary from 2 years in the worst-case scenario to nearly 6 years in the best-case scenario. In Northern Europe, these gains would vary from 3 years to nearly 6 years, and in Southern Europe, from 2.6 years to nearly 5.5 years. The between-scenario variation in women is similar to what is observed in men. Overall, compared to the business-as-usual scenario, the two response scenarios and the best-case scenario present higher gains in life expectancy while the “Desolation Health” and the worst-case scenario have smaller gains, which is consistent across the three regions. Evidence for prolonged life expectancy combined with the result on disease incidence suggest that as people live longer, the number of new NCD cases will increase significantly.

**Fig 7:**
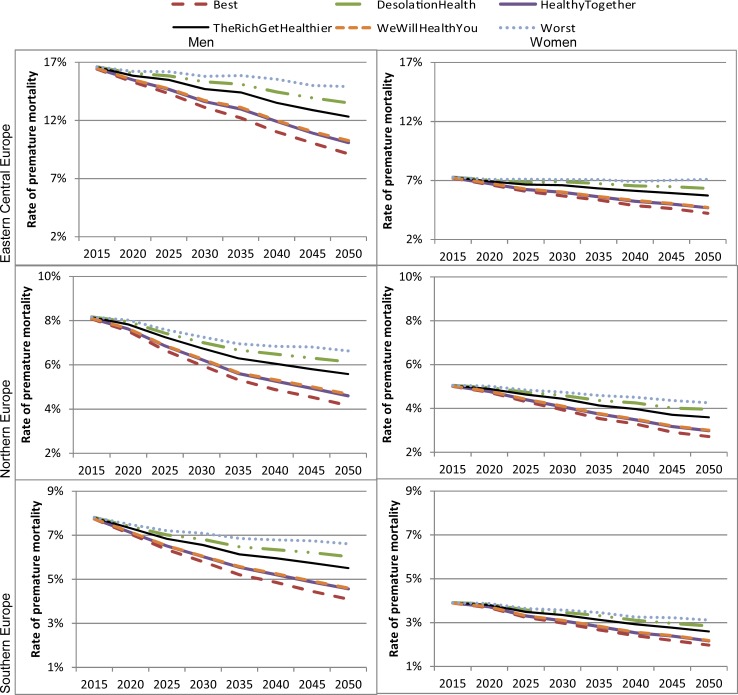
Premature mortality rate from NCDs by 2050 under different scenarios, by gender and region. Source: Authors’ estimates based on microsimulation model, April 2018.

Fig 7 presents the possible evolution of premature mortality, by gender and region. The rate of premature mortality is expected to decline in all scenarios to varying degrees. Changes in premature mortality under the “Desolation Health” and worst-case scenario are markedly worse than in other scenarios, particularly in Central Eastern Europe. For instance, the female premature death rate in Eastern-Central Europe decreases by 21% from 2015 to 2050 in “The Rich Get Healthier” scenario, while it decreases by 13% in “Desolation Health” and by 2% in the worst case scenario.

## Discussion

To the best of our knowledge, this study combines for the first time foresight and modelling techniques to predict the future burden of NCDs in the Europe. Based on the likely evolution of societal trends that impact health and NCDs, four scenarios were designed to predict alternative future health outcomes: one business-as-usual scenario, two response scenarios and one pessimistic scenario. In addition, best-case and worst-case scenarios were also included. Each scenario was associated with an evolution of RFs that predicts the burden of NCDs by 2050.

Results show that the disease burden is likely to grow as people live longer and as the population ages. In optimistic scenarios (“We will Health you” and “Healthy Together”), diseases will appear in later ages, while in more pessimistic scenarios disease will impair working-age people. Life expectancy is projected to grow in all scenarios, but with differences by up to 4 years across scenarios in certain population groups. Premature mortality from NCDs will be reduced in more optimistic scenarios, but might stagnate in pessimistic scenarios in Central-Eastern Europe. Differences across the three studied regions–in particular, poorer health outcomes in Central Eastern Europe- may reflect differences in demographic, economic and political evolution, among others.

Comparisons across different scenarios suggest two important results: first, population ageing is an important factor shaping the future burden of NCDs; and second, that changes in RFs is a key factor explaining the spread of NCDs. Comparison across scenarios shows that improved lifestyles (as seen under the two response scenarios “We Will Health You” and “Healthy Together”) can lead to important reductions in the burden of disease as well as lead to sizeable gains in life expectancy.

### Policy context and implications

Compared to the business-as-usual scenario, the two response scenarios “Healthy Together” and “We Will Health You” which encompass optimistic economic, environmental and social perspectives, would alleviate the burden of disease and increase life expectancy in all three European regions. Conversely, deteriorated societal trends described in the “Desolation Health” scenario would lead to worsened health outcomes. For instance, the prevalence of diabetes under the “Desolation Health” scenario is projected to exceed the level of the worst-case projection, which is in line with expert opinion regarding the growth rate of obesity.

Public health policies aimed at reducing behavioural RFs for NCDs are necessary to achieve the most optimistic scenarios mentioned in this study. Policies beyond the health sector such as education, labour, housing, environment, agriculture, and transport, are also desirable to improve the living environment of people and thus population health.

Premature mortality from NCDs would decline in most scenarios, albeit to varying degrees. Under the business-as-usual scenario, the expected reduction in premature mortality between 2015 and 2030 would range from 9% to 17% depending on gender and region. Nevertheless, this figure would not meet the SDG target to reduce premature mortality from NCDs by one-third between 2010 and 2030 [25]. Findings for the two optimistic scenarios suggest a similar conclusion. This reinforces the need for cross-sectoral policy actions beyond the health sector to tackle unhealthy behavioural RFs.

### Limitations

At least four limitations can be discussed. First, estimates of premature mortality presented herein are below WHO estimates because the model does not capture all types of cancers and CVDs, and accounts for a fraction of the deaths attributable to the major NCDs. Second, the projections of the different scenarios rely on the same demographic projection data, which assumes a common trend in life expectancy aside from the contribution of RFs. The model does not account for any decrease in access to healthcare services that could occur in the “Desolation Health” scenario and that would affect health outcomes and life expectancy. Besides, the unhealthy lifestyles simulated in “Desolation Health” and the worst-case scenarios are not sufficient to curb historical trends in increasing life expectancy. As a result, life expectancy is forecasted to increase in all projections, even in those particularly pessimistic ones. Third, the integration of the foresight and modelling techniques assumes that the pivot between both techniques lies in the RFs. For instance, under the “Desolation Health” scenario, deteriorated social, environmental and economic situations will lead to worsened lifestyles which in turn cause poor outcomes in morbidity and mortality. This approach does not explicitly account for the possible effect of scenarios on healthcare access, and the potential development of new technologies, breakthrough and medicine that may directly influence the treatment of NCDs–although these evolutions might already be captured in some ways by decreasing mortality trends in population projections. Fourth, the quality of the scenarios depends specifically on prior information and competency of the experts taking part in the consultation. An attempt to overcome this limitation was to include quantitative predictive trends (best and worst-case scenarios) in addition to the four narrative scenarios.

## Supporting information

S1 Appendix Additional details on the integrated scenario building method. (DOCX)

S2 Appendix Additional details on the model. (DOCX)

S1 Fig Incidence of COPD and dementia under different scenarios, 2015 and 2050, by region. (DOCX)

S2 Fig Prevalence of people with one or more diseases under the business-as-usual scenario (The Rich get Healthier), three zones, 2050. (DOCX)

## Abbreviations

CVD: Cardiovascular diseases
COPD: Chronic Obstructive Pulmonary Disease
FRESHER: FoResight and Modelling for European Health policy and Regulation
GBD: Global Burden of Diseases
IHME: Institute for Health Metrics and Evaluation
NCDs: non-communicable diseases
OECD: Organisation for Economic Cooperation and Development
RFs: Risk factors
WHO: World Health Organization

## References

[1] Garg CG, Evans DB What is the impact of Non Communicable diseases on National Health Expenditures: a synthesis of available data. WHO Discussion Paper No. 3. 2011. WHO, Geneva. Available from: https://www.who.int/healthsystems/NCDdiscussionpaper3.pdf

[2] Muka T, Imo D, Jaspers L, Colpani V, Chaker L, van der Lee SJ, et al The global impact of non-communicable diseases on healthcare spending and national income: a systematic review. Eur J Epidemiol. 2015 4;30(4):251–77. 10.1007/s10654-014-9984-2 25595318

[3] World Health Organization. Global strategy for the prevention and control of noncommunicable diseases (WHAA53/14). Geneva: World Health Organization; 2000.

[4] World Health Organization. Preventing chronic diseases: a vital investment. Geneva: World Health Organization; 2005. http://www.who.int/chp/chronic_disease_report/full_report.pdf

[5] World Health Organization. Equity, social determinants and public health programmes. Geneva: World Health Organization; 2010.

[6] World Health Organisation. Sixty-Sixth World Health Assembly. Follow-up to the Political Declaration of the High-level Meeting of the General Assembly on the Prevention and Control of Non-communicable Diseases. 27 May 2013. Accessed on 6 April 2018. Available from: http://apps.who.int/gb/ebwha/pdf_files/WHA66/A66_R10-en.pdf?ua=1

[7] United Nations, 2015. Transforming our World: The 2030 Agenda for Sustainable Development. Accessed on 11 February 2020. Available from: https://sustainabledevelopment.un.org/post2015/transformingourworld/publication

[8] Kontis V, Mathers C, Bonita R, Stevens G, Rehm J, Shield K, et al Regional contributions of six preventable risk factors to achieving the 25x25 non-communicable disease mortality reduction target: a modelling study. The Lancet Global Health. 2015;3, e746–e757. 10.1016/S2214-109X(15)00179-5 26497599

[9] Li Y, Zeng X, Liu J, Liu Y, Liu S, Yin P, et al Can China achieve a one-third reduction in premature mortality from non-communicable diseases by 2030? BMC Medicine. 2017;15(1):132 10.1186/s12916-017-0894-5 28693510 PMC5504650

[10] González-Pier E, Barraza-Lloréns M, Beyeler N, Jamison D, Knaul F, Lozano R et al. Mexico's path towards the Sustainable Development Goal for health: an assessment of the feasibility of reducing premature mortality by 40% by 2030. Lancet Glob Health. 2016 10;4(10):e714–25. 10.1016/S2214-109X(16)30181-4 Epub 2016 Aug 30. 27596038 PMC5024342

[11] Cobiac L, Scarborough P. Translating the WHO 25×25 goals into a UK context: the PROMISE modelling study. BMJ Open. 2017;7:e012805 10.1136/bmjopen-2016-012805 28377390 PMC5387932

[12] Webber L, Divajeva D, Marsh T, McPherson K, Brown M, G et al The future burden of obesity-related diseases in the 53 WHO European Region countries and the impact of effective interventions: a modelling study. BMJ Open. 2014 7 25;4(7):e004787 10.1136/bmjopen-2014-004787 25063459 PMC4120328

[13] Foreman KJ, Marquez N, Dolgert A, Fukutaki K, Fullman N, McGaughey M et al. Forecasting life expectancy, years of life lost, and all-cause and cause-specific mortality for 250 causes of death: reference and alternative scenarios for 2016–40 for 195 countries and territories. Lancet. 2018; 392:2052–90. 10.1016/S0140-6736(18)31694-5 30340847 PMC6227505

[14] United Nations. World Population Prospects—Population Division—United Nations. Retrieved 01 01, 2016, from https://population.un.org/wpp/

[15] Human Mortality Database. Retrieved 03 29, 2016, from http://www.mortality.org/

[16] GBD 2016 Disease and Injury Incidence and Prevalence Collaborators, Vos T, Abajobir A, Abate K, Abbafati C, Abbas K, Abd-Allah F, et al Global, regional, and national incidence, prevalence, and years lived with disability for 328 diseases and injuries for 195 countries, 1990–2016: a systematic analysis for the Global Burden of Disease Study 2016. Lancet (London, England). 2017:390(10100):1211–1259. 10.1016/S0140-6736(17)32154-2 PMC5605509 28919117

[17] WHO, 2018, Global Health Observatory Data Repository, Indicator on Average daily intake in grams of alcohol, by country. Accessed on 21 December 2017 at: http://apps.who.int/gho/data/node.main.A1037?lang=en

[18] NCD Risk Factor Collaboration (NCD-RisC), Abarca-Gomez L, Abdeen Z, Hamid Z, Abu-Rmeileh N, Acosta-Cazares B, Acuin C, et al Worldwide trends in body-mass index, underweight, overweight, and obesity from 1975 to 2016: a pooled analysis of 2416 population-based measurement studies in 128·9 million children, adolescents, and adults. Lancet (London, England). 2017;390(10113):2627–2642. 10.1016/S0140-6736(17)32129-3 PMC5735219 29029897

[19] UK Government Office for Science. 2017: The Futures Toolkit, Tools for Futures Thinking and Foresight Across UK Government. Accessed on 6 July 2018. Available from: https://assets.publishing.service.gov.uk/government/uploads/system/uploads/attachment_data/file/674209/futures-toolkit-edition-1.pdf

[20] Giesecke S, Van der Gießen A, Elkins S. (Eds.) (2012). The role of forward-looking activities for the governance of Grand Challenges. Insights from the European Foresight Platform. Vienna.

[21] Cagnin C, Johnston R, Giesecke S. Foresight contribution to grand challenges and participative governance in different cultural settings. Technological Forecasting & Social Change. 2015; 101:182–184. 10.1016/j.techfore.2015.11.020

[22] Cecchini, Devaux M, Sassi F. Assessing the impacts of alcohol policies: A microsimulation approach. OECD Health Working Paper No. 80. Paris: OECD; 2015.

[23] Devaux M, Lerouge A, Ventelou, Goryakin Y, Feigl A, Vuik S et al Assessing the potential outcomes of achieving the World Health Organization global non-communicable diseases targets for risk factors by 2025: is there also an economic dividend? Public Health. 2019. 169:173–179. 10.1016/j.puhe.2019.02.009 30876722

[24] Cecchini M, Cortaredona S, Devaux M, Elfakir A, Goryakin Y, Lerouge A et al., Scientific paper on the methodology, results and recommendation for future research, FRESHER report Deliverable 5.3, December 2017. Accessed on 13 June 2018. Available from: https://www.foresight-fresher.eu/content/uploads/2018/03/d5-3-scientific-paper-on-the-methodology-results-and-recommendation-for-future-research.pdf

[25] UN United Nations and General Assembly. Political declaration of the high-level meeting of the general assembly on the prevention and control of non-communicable diseases. 24 January 2012. Accessed on 6 April 2018. Available from: http://www.who.int/nmh/events/un_ncd_summit2011/political_declaration_en.pdf

