# Supplementary material for: How will the main risk factors contribute to the burden of non-communicable diseases under different scenarios by 2050? A modelling study
Source: PLoS One. 2020 Apr 29;15(4):e0231725. doi: 10.1371/journal.pone.0231725 (PMC7190114; doi:10.1371/journal.pone.0231725)
Supplement: S1 Appendix — (DOCX) [file pone.0231725.s001.docx]

**Supporting Information Appendix for paper entitled “How will the main risk factors contribute to the burden of non-communicable diseases under different scenarios by 2050? A modelling study”**

S1 Appendix. Additional details on the FRESHER Scenario building

1. Additional details on the integrated scenario building method

FRESHER (“FoResight and Modelling for European Health policy and Regulation”) is an interdisciplinary research project funded by the European Union’s Horizon 2020 research and innovation programme under grant agreement No 643576 for a duration of 36 months (2015-2018). One output of this project was the four future-looking scenarios (‘FRESHER Scenarios’), written in the form of storytelling narratives, describing possible impacts of the combination of identified trends on the future of health and non-communicable diseases (NCDs). The full narrative description of the four scenarios is available at: <https://www.foresight-fresher.eu/fresher-project-results/scenario-building-process/>

Scenarios represent “possible set of future conditions- describing one or more visions/stories of possible future, highlighting the discontinuities from the present, revealing the choice available and their potential consequences^[[1]](#endnote-1)^”.

In line with current practice, FRESHER Scenarios have been developed for the EU countries with the target year 2050. This time frame is essential to be able to envisage paradigmatic changes in terms of policies and behavioural change. The FRESHER Scenarios combined an explorative approach with a normative prospective. Explorative approaches imply a desire to know what the future will be like, so as to adjust to expected change, while normative approaches imply a belief that futures-oriented planning can change development paths.

FRESHER scenarios were developed according to the intuitive logics foresight school^^[[2]](#endnote-2)^^, which originated with RAND and is now strongly associated with Shell Oil and the Global Business Network. FRESHER Scenarios Building process has emphasised the participatory dimension: four-hundred experts and stakeholders, with different backgrounds within and beyond health fields, were involved throughout all the steps of the process, in two surveys and nine workshops (figure below).


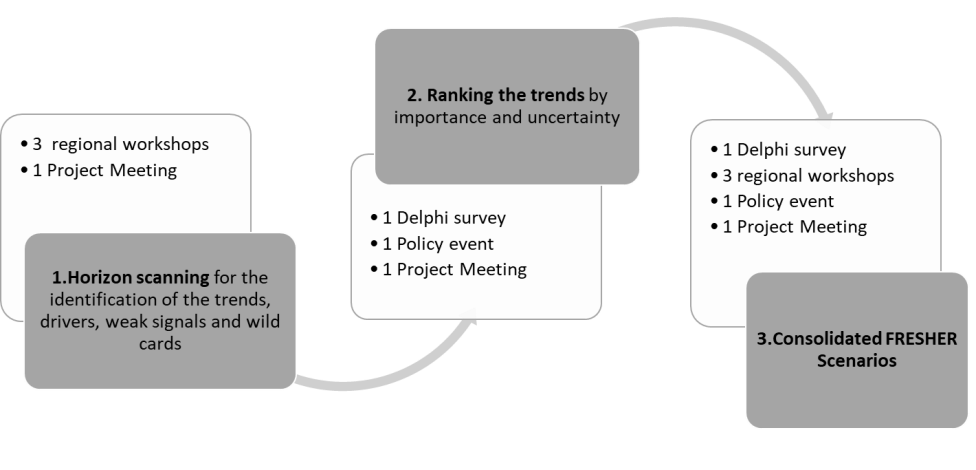


The Horizon Scanning and Driver Identification phase has identified the health related short, medium and long-term trends, drivers, wild cards and weak signals in the future of health and non-communicable diseases (NCDs). The project team conducted an extensive literature review on trends and drivers of NCDs, complemented by the organisation of three stakeholders’ workshops (Vienna, Brussels, Lisbon).

Eight key trends, represented in the table below, were selected by aggregating and fine-tuning the drivers emerged as most important for the future of NCDs in the Horizon Scanning workshops. In order to validate the selection, the project team categorized the trends according the following categories: 1) socio-demographic; 2) technological; 3) economic; and 4) environmental. For each of the trends, an indicator was also agreed to understand its past development and look its possible logic evolution. A dedicated survey “What will impact your health the most?” was then launched with the aim of assessing the relative importance of the trends and their degree of predictability/uncertainty in a long-term future (2050).

| Critical uncertainty | Indicator |
| --- | --- |
| Equity | GINI index |
| Economic pattern and technological change | Growth and living standards |
| Innovation in medicine | Cost-effectiveness |
| Citizens empowerment | Access to information for prevention |
| Climate change and low carbon economy | Frequency of extreme events and heat waves |
| Demographic change | Healthy life years |
| Urbanisation | Exposure to health pollution |
| Agriculture and global food chains | Healthy diets |

In investigating the trends development pathways, two overarching trends were ultimately recognised as fundamental: the dynamics of equity and the progress towards a low carbon economy. Both trends were considered to: i) have a high impact on the future of NCDs; ii) be highly uncertain; iii) strongly influence all other trends. The analysis on how the evolution of these two trends might be combined with the other 6 trends development at 2050 conducted to select four Scenarios. These so-called ‘FRESHER Scenarios’ were written in the form of 'storytelling' narratives describing possible impacts of the identified trends combination on the future of health and NCDs.

The FRESHER survey “How healthy will your future be?” was then disseminated to elicit stakeholders’ contributions in order to validate the Scenarios and liaise the qualitative work with the micro-simulation model. In addition, three regional workshops were held to downscale the Scenarios (Warsaw, Coruna, Helsinki).

1. **Description of the four FRESHER scenarios**

The four FRESHER Scenarios are briefly described below, and a more detailed description is available on a dedicated website: <https://www.foresight-fresher.eu/fresher-project-results/>.

**The Rich Get Healthier** - staying the course: In this scenario, freedom and meritocracy are the pillars of societal structure. Market forces are dominant and a ‘light government’ guarantees their functioning. European states have tended to privatise the health-care sector to reduce public debt and have deregulated labour to revitalise the economy. Health is now just like many other services: potentially available but expensive. Human health and lifestyle are left to individuals’ choices and capacities. The more you can afford it, the better treatment you get, thanks to expensive medical innovations including new-generation biomedical devices. The global protection of the environment is ensured by pricing it. It is a socio-economic system where most economies are decarbonised and climate change is now under control. However, global governance focuses on achieving results, without considering level of inclusion and equity of the solutions pursued.

**We will Health you** - sustained innovation for a healthy workforce: In this second scenario, today’s priority is to maintain a healthy workforce, for the continuation of economic productivity and for ensuring the sustainability of the healthcare systems. Thanks to big data, public and private investments effectively influence citizens’ behaviour towards healthy lifestyles. Employers provide healthy working environments and care services provided. Fair labour legislation is implemented to give workers money, time and knowledge to take better care of their health. The top down approach is ensured by ambient 24/7 surveillance and implanted chips for affordable early diagnostics, tele-medicine and tailor-made treatment. The new era of economic growth and social progress focused on delivering more to everyone, with environmental sustainability seriously overlooked. Increasing members of economic and environmental migrants are allowed in implementation of following strict immigration policy and by considering only their skills and possible contributions to the EU economy’s growth.

**Healthy together -** promoting health and well-being for all: The main priority in society is to promote health and well-being for all. Governments, the private sector and citizens’ networks collaborate closely to develop solutions to promote quality of life, healthy opportunities and efficient care. When governments take the lead, citizen participation is ensured throughout the policy making process, to promote equity, sustainability and human health in all policies. There is high value in leisure, sense of community and nature. Fair incomes level up living conditions, ensuring better standards to all. A new socio-economic pattern provides for the means to take better care of one’s own health but also to care about others through informal networks and community engagement Recycling and sharing practices lower the focus on productivity and pressure on the environment.

**Desolation Health -** The European model has declined and European governance, shared values and the common market have fallen apart amidst economic stagnation and recession. To gain some legitimacy, national governments cooperate with different stakeholders for short-sighted policies and do not consider health implications. Economic stagnation has led many countries to gradually reduce number of people that can avail public services, increase user charges for services and limit the number of public health providers. Health shocks -defined as unpredictable illnesses which diminish health status- increase, and innovative medicines, focusing on quick-fix solutions, and treatments remain hardly affordable for European governments and citizens. The deterioration of living standards undermines community values and lead to tensions among citizens and mistrust of policies. Citizens suffer the consequences of climate change as international agreements have stalled for decades.

1. EC FOR –LEARN on line foresight guide <http://forlearn.jrc.ec.europa.eu/guide/0_home/index.htm> [↑](#endnote-ref-1)
2. Bradfield R. et al (2005), “The origins and evolution of scenario techniques in long range business planning”, Futures 37:795–812 [↑](#endnote-ref-2)
