# Supplementary material for: How will the main risk factors contribute to the burden of non-communicable diseases under different scenarios by 2050? A modelling study
Source: PLoS One. 2020 Apr 29;15(4):e0231725. doi: 10.1371/journal.pone.0231725 (PMC7190114; doi:10.1371/journal.pone.0231725)
Supplement: S2 Appendix — (DOCX) [file pone.0231725.s002.docx]

**Supporting Information Appendix for paper entitled “How will the main risk factors contribute to the burden of non-communicable diseases under different scenarios by 2050? A modelling study”**

S2 Appendix. Additional details on the model

The general framework of the model is presented in Cecchini et al (2017)^[[1]](#endnote-1)^ and Devaux et al (2019)^[[2]](#endnote-2)^. A full documentation of the model structure is made freely available online, including a report on the demography module^[[3]](#endnote-3)^ and a report on the diseases and risk factors module^i^.

The data sources for diseases and risk factors (RFs) exposure (smoking and physical inactivity) use the IHME Global Burden of Diseases (GBD) 2016^[[4]](#endnote-4)^. Alcohol consumption is modelled using the level of alcohol consumption in WHO Global Health Observatory^[[5]](#endnote-5)^ combined with the distribution parameters from IHME GBD 2015. Population exposure to obesity and blood pressure uses data from NCD-RisC^[[6]](#endnote-6)^.

In the model, the following definition of the RFS are used:

- Smoking refers to current smoking.
- Alcohol consumption includes the level of alcohol intake (measured in grams of pure alcohol per day) and the prevalence of binge drinking (having six (five) or more drinks per occasion in the past 12 months for men (women)).
- Obesity is defined as a body mass index above 30 kg/m2.
- Physical inactivity is defined as having below 600 METs. High blood pressure is defined as having a systolic blood pressure higher than 140 mmHg.

Relative risks that link RFs to diseases are determined by gender and age group. Information on relative risks was collected from IHME GBD 2016^^[[7]](#endnote-7)^^, the DYNAMO-HIA model^^[[8]](#endnote-8)^^, and the OECD alcohol model^[[9]](#endnote-9)^.

The model is developed for three regions in European Union countries:

- Central/Eastern Europe (Bulgaria, Poland, Romania, Slovakia, Estonia, Hungary, Latvia and Lithuania);
- Northern Europe (Austria, Belgium, Czech Republic, Denmark, Finland, Germany, Ireland, Luxembourg, the Netherlands, Sweden and United Kingdom);
- Southern Europe (Croatia, Cyprus, France, Greece, Italy, Malta, Portugal, Slovenia and Spain).

**Expert’s consultation**

An expert’s consultation was carried out in 2017 to survey health experts about the evolution of five key behavioural risk factors -at the 2050 horizon- under each of FRESHER Scenarios. Given their expertise and the description of the four FRESHER scenarios they were provided with, the experts evaluated the level of the risk factors in 2050 for each of the four scenarios. We collected responses from 91 health experts (most of them from academia). Table 1 shows the mean values across the 91 expert’s responses. For instance, obesity rate is 27% in Europe in 2015. On average across the 91 expert’s responses, obesity rate will increase to 35.4% in 2050 in the ‘The rich gets healthier’ scenario.

Table 1. Experts’ evaluation of future trends in risk factors (mean)

| **Risk Factors** | **Average (2015)** | **Worst case (Minimum value observed in 2015)** | **Best case (Maximum value observed in 2015)** | **The rich get healthier (2050)** | **Healthy together (2050)** | **We will health you (2050)** | **Desolation health  (2050)** |
| --- | --- | --- | --- | --- | --- | --- | --- |
| Obesity (%) | *27* | *23* | *32* | **35.4** | **24.4** | **25.4** | **38.6** |
| Phys inactivity (%) | *32* | *12* | *55* | **35.8** | **28.8** | **30.4** | **38.0** |
| Smoking (%) | *23* | *12* | *35* | **21.1** | **15.3** | **15.5** | **25.4** |
| Alcohol (g/day) | *10.72* | *4.51* | *17.52* | **12.63** | **9.57** | **9.54** | **14.98** |
| High Blood Pressure (% with SBP >140 mmHg) | *31* | *22* | *44* | **32.2** | **24.7** | **25.2** | **36.9** |

Note: Green (blue) colour means the level has decreased (increased) compared to 2015. The levels of alcohol consumption as presented in this table are originally taken from the IHME data, however it has been rescaled in the model to match the OECD average per capita level.

Source: IHME 2015 data in European countries, and Expert’s consultation for 2050.

**Sources of data used in the model**

**S1 Table. Data source for the model**

| **Model module** | **Parameter** | **Source** |
| --- | --- | --- |
| Demography | Population projection | United Nations. World Population Prospects - Population Division - United Nations [dataset]. Accessed on January 2018. Available from: <https://population.un.org/wpp/> |
|  | Mortality | Human Mortality Database [dataset]. Accessed on 29 March 2016. Available from: <http://www.mortality.org/> |
| Diseases | Disease incidence, prevalence, remission and fatality | GBD 2016 Disease and Injury Incidence and Prevalence Collaborators, Vos T, Abajobir A, Abate K, Abbafati C, Abbas K, Abd-Allah F, et al. Global, regional, and national incidence, prevalence, and years lived with disability for 328 diseases and injuries for 195 countries, 1990-2016: a systematic analysis for the Global Burden of Disease Study 2016. The Lancet. 2017;390(10100):1211-1259. |
| Risk Factors | Smoking, physical inactivity, and salt intake | GBD 2016 Disease and Injury Incidence and Prevalence Collaborators, Vos T, Abajobir A, Abate K, Abbafati C, Abbas K, Abd-Allah F, et al. Global, regional, and national incidence, prevalence, and years lived with disability for 328 diseases and injuries for 195 countries, 1990-2016: a systematic analysis for the Global Burden of Disease Study 2016. The Lancet. 2017;390(10100):1211-1259. |
|  | Alcohol | World Health Organization, Global Health Observatory Data Repository, Average daily intake in grams of alcohol, by country [dataset]. Accessed on 21 December 2017. Available from: <http://apps.who.int/gho/data/node.main.A1037?lang=en>  GBD 2015 Risk Factors Collaborators, Forouzanfar M, Afshin A, Alexander L, Anderson H, Bhutta Z, Biryukov S, et al. Global, regional, and national comparative risk assessment of 79 behavioural, environmental and occupational, and metabolic risks or clusters of risks, 1990–2015: a systematic analysis for the Global Burden of Disease Study 2015. The Lancet. 2016;388(10053):1659-1724 |
|  | Obesity and blood pressure | NCD Risk Factor Collaboration (NCD-RisC). Worldwide trends in body-mass index, underweight, overweight, and obesity from 1975 to 2016: a pooled analysis of 2416 population-based measurement studies in 128·9 million children, adolescents, and adults. The Lancet. 2017:390(10113):2627-2642. |
|  | Relative risks | Gakidou E, Afshin A, Abajobir A, Abate K, Abbafati C, Abbas K, et al. Global, regional, and national comparative risk assessment of 84 behavioural, environmental and occupational, and metabolic risks or clusters of risks, 1990–2016: a systematic analysis for the Global Burden of Disease Study 2016. The Lancet. 2017;390(10100):1345-1422.  Lhachimi S, Nusselder W, Smit H, van Baal P, Baili P, Bennett K, et al. Dynamo-HIA-a dynamic modeling tool for generic health impact assessments. PLoS ONE. 2012;7(5): e33317.  Cecchini M, Devaux M, Sassi F. Assessing the impacts of alcohol policies: A microsimulation approach. OECD Health Working Paper No. 80, Paris. 2015. |

Note: see definition of the parameter above in the main text.

1. Cecchini M, Cortaredona S, Devaux M, Elfakir A, Goryakin Y, Lerouge A et al., Scientific paper on the methodology, results and recommendation for future research, FRESHER report Deliverable 5.3, December 2017. Accessed on 13 June 2018. Available from : <https://www.foresight-fresher.eu/content/uploads/2018/03/d5-3-scientific-paper-on-the-methodology-results-and-recommendation-for-future-research.pdf> [↑](#endnote-ref-1)
2. Devaux M, Lerouge A, Ventelou, Goryakin Y, Feigl A, Vuik S et al. Assessing the potential outcomes of achieving the World Health Organization global non-communicable diseases targets for risk factors by 2025: is there also an economic dividend? Public Health. 2019. 169:173-179. doi:10.1016/j.puhe.2019.02.009. [↑](#endnote-ref-2)
3. Aubrecht C. et al (2016), Validated European Health Policy Model software and Documentation, FRESHER report Deliverable 5.1, July 2016. Accessed on 13 July 2018. Available from :<https://www.foresight-fresher.eu/content/uploads/2018/03/d5-1-validated-european-health-policy-model-software-and-documentation-corrected.pdf> [↑](#endnote-ref-3)
4. GBD 2016 Disease and Injury Incidence and Prevalence Collaborators, T., Abajobir, A., Abate, K., Abbafati, C., Abbas, K., Abd-Allah, F., et al. (2017). Global, regional, and national incidence, prevalence, and years lived with disability for 328 diseases and injuries for 195 countries, 1990-2016: a systematic analysis for the Global Burden of Disease Study 2016. Lancet (London, England), 390(10100), 1211-1259. [↑](#endnote-ref-4)
5. WHO, 2018, Global Health Observatory Data Repository, Indicator on Average daily intake in grams of alcohol, by country. Accessed on 21 December 2017 at: <http://apps.who.int/gho/data/node.main.A1037?lang=en> [↑](#endnote-ref-5)
6. NCD Risk Factor Collaboration (NCD-RisC), L., Abdeen, Z., Hamid, Z., Abu-Rmeileh, N., Acosta-Cazares, B., Acuin, C., et al. (2017). Worldwide trends in body-mass index, underweight, overweight, and obesity from 1975 to 2016: a pooled analysis of 2416 population-based measurement studies in 128·9 million children, adolescents, and adults. Lancet (London, England), 390(10113), 2627-2642. [↑](#endnote-ref-6)
7. Gakidou E, Afshin A, Abajobir A, Abate K, Abbafati C, Abbas K, et al. Global, regional, and national comparative risk assessment of 84 behavioural, environmental and occupational, and metabolic risks or clusters of risks, 1990–2016: a systematic analysis for the Global Burden of Disease Study 2016. The Lancet. 2017;390(10100):1345-1422. [↑](#endnote-ref-7)
8. Lhachimi S, Nusselder W, Smit H, van Baal P, Baili P, Bennett K, et al. Dynamo-HIA-a dynamic modelling tool for generic health impact assessments. PLoS ONE. 2012;7(5): e33317 [↑](#endnote-ref-8)
9. Cecchini, M., Devaux, M., Sassi, F. (2015). Assessing the impacts of alcohol policies: A microsimulation approach. OECD Health Working Paper No. 80, Paris. [↑](#endnote-ref-9)
