# Supplementary material for: How will the main risk factors contribute to the burden of non-communicable diseases under different scenarios by 2050? A modelling study
Source: PLoS One. 2020 Apr 29;15(4):e0231725. doi: 10.1371/journal.pone.0231725 (PMC7190114; doi:10.1371/journal.pone.0231725)
Supplement: S1 Fig — (DOCX) [file pone.0231725.s003.docx]

**Supporting Information Appendix for paper entitled “How will the main risk factors contribute to the burden of non-communicable diseases under different scenarios by 2050? A modelling study”**

S1 Fig. Incidence of COPD and dementia under different scenarios, 2015 and 2050, by region


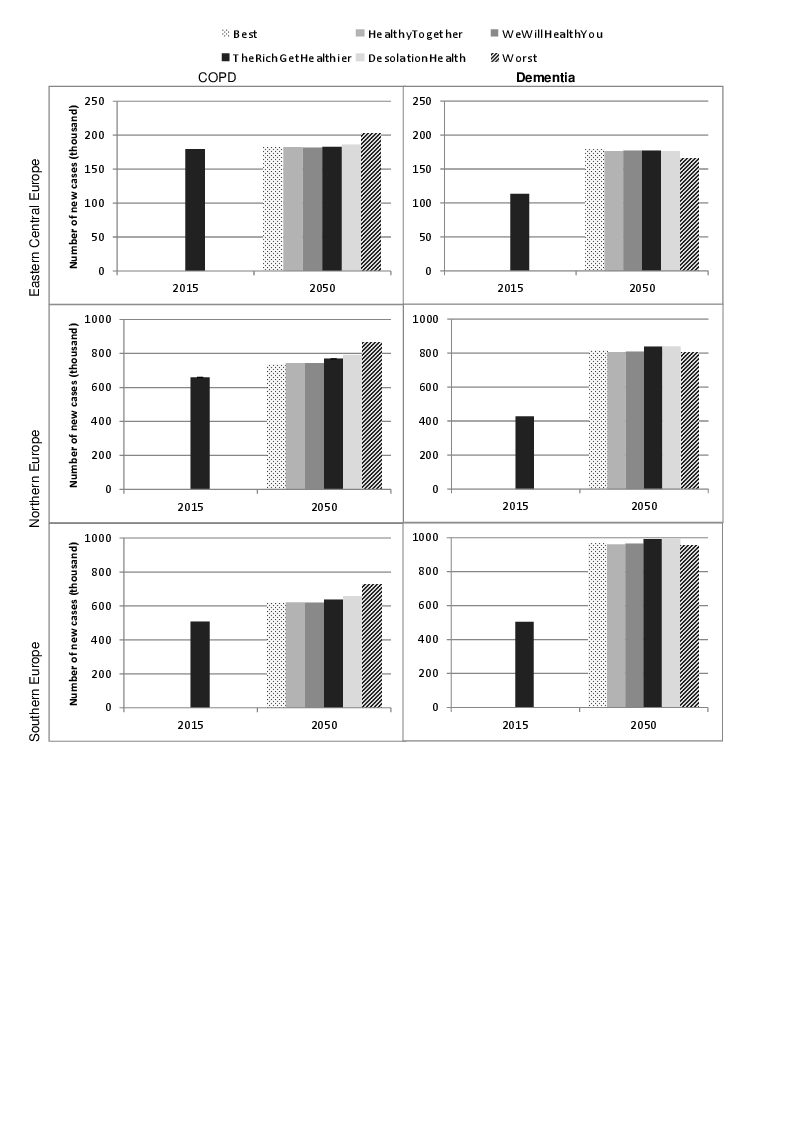


Source: Authors’ estimates based on microsimulation model, April 2018.
