# Supplementary material for: How will the main risk factors contribute to the burden of non-communicable diseases under different scenarios by 2050? A modelling study
Source: PLoS One. 2020 Apr 29;15(4):e0231725. doi: 10.1371/journal.pone.0231725 (PMC7190114; doi:10.1371/journal.pone.0231725)
Supplement: S2 Fig — (DOCX) [file pone.0231725.s004.docx]

**Supporting Information Appendix for paper entitled “How will the main risk factors contribute to the burden of non-communicable diseases under different scenarios by 2050? A modelling study”**

S2 Fig. Prevalence of people with one or more diseases under the business-as-usual scenario (The Rich get Healthier), three zones, 2050

Panel A. Central-Eastern Europe

Panel B. Northern Europe

Panel C. Southern Europe

Source: Authors’ estimates based on microsimulation model, April 2018.
